# Effects of Inorganic Salts on Curdlan Production and Structural Properties

**DOI:** 10.3390/gels11050313

**Published:** 2025-04-23

**Authors:** Xinyi Zhu, Bowei Yao, Siyang Yue, Zhongyi Chang, Xuexia Yang, Hongliang Gao

**Affiliations:** 1 College of Biological Science and Medical Engineering, Donghua University, Shanghai 201620, China; zhuxinyi0820@163.com; 2 School of Life Sciences, East China Normal University, Shanghai 200241, China; siyangyue2000@163.com (S.Y.); zychang@bio.ecnu.edu.cn (Z.C.); hlgao@bio.ecnu.edu.cn (H.G.); 3 NingXia Academy of Metrology & Quality Inspection, Yinchuan 750001, China

**Keywords:** curdlan, inorganic salts, gel strength, texture, molecular weight

## Abstract

This research investigates the influence of inorganic salts on curdlan production, gel strength, molecular weight (Mw), and texture properties, to provide insights into optimizing fermentation conditions. Five different inorganic salts were individually incorporated into the fermentation medium to assess their impact. The results showed that FeCl_3_ significantly reduced curdlan yield, Mw, and gel quality, indicating its unsuitability for curdlan fermentation. FeSO_4_ at 0.01% enhanced yield, gel strength, and texture properties, such as springiness and chewiness, though higher concentrations had adverse effects. MnCl_2_ exhibited limited impact on yield, with low concentrations notably decreasing gel strength, hardness, springiness, and chewiness. CoCl_2_ was the most effective additive, with a concentration of 0.002% achieving optimal results for yield, Mw, gel strength, and texture, though higher levels diminished these benefits. ZnCl_2_ at 0.04% improved gel strength, chewiness, and Mw but had minimal effect on other properties. A correlation (R^2^ = 0.5064) was observed between Mw and gel strength, indicating Mw’s critical role in curdlan’s mechanical properties. Overall, CoCl_2_ and FeSO_4_ at specific concentrations demonstrated potential for enhancing curdlan quality and offered practical insights for tailoring fermentation processes to achieve desired properties in industrial applications.

## 1. Introduction

**Figure 1:**
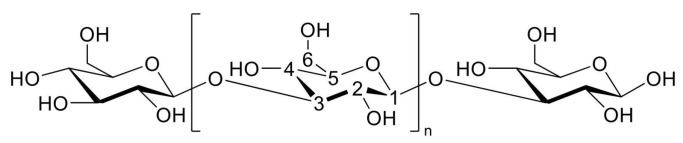
Molecular structure of curdlan.

Curdlan, a non-ionic extracellular β-1,3-glucan homopolymer, is biosynthesized via microbial fermentation using *Agrobacterium* spp. (e.g., *Agrobacterium* sp. ATCC 31749); the molecular formula of curdlan is (C_6_H_10_O_5_)_n_, and its structure is shown in Figure 1 [1]. Its unique ability to form thermoreversible and thermoirreversible gels under varying thermal conditions makes it highly valuable for texture modification in food products and emulsion stabilization [2]. Additionally, curdlan functions as a stabilizer and can mimic the texture of fat [3], meat [4], and seafood [5]. Beyond its applications in food science, curdlan and its derivatives exhibit notable immune-regulatory properties, making them potential candidates as vaccine adjuvants [6]. Aminated curdlan derivatives, particularly PEGylated adenosine-valeric acid-curdlan (pAVC) polymers, have demonstrated potential as carriers for the tumor-specific transportation of nucleic acid drugs due to their enhanced biocompatibility and targeting efficiency [7]. Furthermore, curdlan has been incorporated into advanced drug delivery systems, such as curdlan-grafted poly-N-isopropylacrylamide/montmorillonite nanocomposites, for which reinforced alginate beads were developed to achieve sustained release and improved bioavailability of Erlotinib HCl, enabling precision delivery to chemoresistant triple-negative breast cancer (TNBC) cells [8]. Curdlan has been reported to inhibit tumor growth, activate leukocytes, and stimulate cytokine production in humans, further highlighting its therapeutic potential [9].

To meet the growing demand for curdlan, it is essential to optimize production processes, including medium formulation and the overall fermentation process. Several factors affect curdlan fermentation, such as dissolved oxygen (DO) [10], pH [11], and the composition of the fermentation medium [12]. Studies have shown that increasing DO from 15% to 60% enhances curdlan yield by 80% in *Agrobacterium* sp. ATCC 31749 [13]. Efficient cell growth requires an initial neutral pH of 7.0, with curdlan production commencing as the pH gradually decreases [14]. When the pH is adjusted to approximately 6 to 7, cells can produce curdlan from sugars under low concentrations of inorganic salts [15]. The components of the fermentation media, including carbon sources [16], nitrogen sources [17], and inorganic salts [18], also play crucial roles. The C/N proportion directly affects the growth rate of *Agrobacterium* sp. [19]. Continuous glucose addition at low concentrations during fermentation reduces the inhibitory effects of excess carbon, significantly improving both curdlan yield and carbon source conversion [20]. Furthermore, curdlan’s structural transition to a hydrogel matrix necessitates an initial macromolecular swelling phase, a process governed by specific effects of inorganic salt ions that perturb solvent structuring and dielectric properties, thereby regulating water mobility and hydration efficiency [21]. Research has also indicated that an increase in salt concentration causes a conformational transition of the glucan from random coils to an ordered structure at concentrations of alkali of less than 0.3 M [22]. Moreover, curdlan production has been linked to the stationary phase of microbial growth, characterized by constant optical density and declining viable counts [23].

In bacterial fermentation, the concentration of inorganic salts in the medium is highly significant for the production of extracellular polysaccharides (EPS). Previous study has indicated that phosphorus influences lipid and carbohydrate uptake, potassium acts as an enzyme cofactor in carbohydrate metabolism, magnesium participates in enzyme function and cell structure, and zinc is essential for primary metabolism and the production of sclerosolan in mineral media [24]. Employing a strategy termed inorganic salt starvation, limiting their supply has been shown to enhance EPS accumulation in bacteria. Studies have shown that NaCl does not stimulate EPS synthesis; conversely, EPS production decreases as its concentration increases. Conversely, CaCO_3_ has been identified as a specific promoter of biopolymer formation [25], underscoring the importance of targeted salt inclusion in fermentation media. Regarding nitrogen sources, the direct addition of ammonium salts to the culture medium has been shown to be more favorable for EPS production compared to nitrate salts. This is because the conversion of nitrate to ammonium requires significant energy expenditure, thereby reducing overall efficiency [26,27]. Inorganic salts also serve as essential cofactors in cellular metabolism, catalyzing biochemical reactions crucial to bacterial growth and EPS biosynthesis [28]. Similarly, EPS have been shown to modulate food matrix properties, significantly influencing the rheology, thermal stability, and microstructure of whole-grain products, such as wheat bran-enriched noodles [29]. Previous studies have demonstrated that inorganic salts, including CaCl_2_, KCl, MgCl_2_, AlCl_3_, Ca (CH_3_COO)_2_, can modulate fermentation processes, storage stability, and structural/textural properties of pickled cucumbers. Notably, calcium ions show exceptional potential in preserving texture and sensory quality, offering practical insights for industrial food processing [30]. Furthermore, modulating EPS biosynthesis through targeted addition of inorganic salts (e.g., Ca^2+^, Fe^3+^) represents a novel strategy for food texture engineering.

The presence of inorganic salts also affects the texture and gel strength of EPS. For example, salt addition to gels affects their hardness and melting temperature, with both properties declining as ionic concentration increases [31]. Furthermore, studies demonstrate that elevated inorganic salt levels significantly reduce gel strength; at 4% concentration, gel strength decreases to nearly one-third of its baseline value [32]. Research indicates that molecular weight is a critical determinant of gel strength; as molecular weight increases, intermolecular forces between polymer molecules strengthen, leading to enhanced hardness and diminished fluidity. Thus, variations in inorganic salts may modulate the molecular weight, gel strength, and overall texture of EPS [33].

To date, no research studies have systematically explored the ramifications of inorganic salts on curdlan production and properties. Understanding the role of inorganic salts and optimizing curdlan properties is essential for enhancing its industrial applications. This study investigated the effects of supplementing the fermentation medium with inorganic salts (FeCl_3_, FeSO_4_, MnCl_2_, CoCl_2_, and ZnCl_2_), examining how salt type and concentration influence curdlan yield and quality. The average molecular weight (Mw) of curdlan was characterized using a gel permeation chromatography (GPC) system to evaluate correlations between Mw and its properties. Additionally, Texture Profile Analysis (TPA) was employed to assess curdlan gel texture, quantifying parameters, such as hardness, springiness, and chewiness, which govern its functional performance. These analyses elucidate the mechanistic effects of inorganic salts on curdlan production and physicochemical traits, providing a foundation for optimizing fermentation processes to enhance product quality.

## 2. Results and Discussion

This study addresses a significant gap in previous studies by examining the effects of diverse inorganic salt ions on curdlan production and its physicochemical properties. Although previous studies have established the role of inorganic salts on the yield and performance of extracellular polysaccharides, specific attention to curdlan has been lacking.

### 2.1. Effect of FeCl_3_ on Curdlan Production and Properties

**Figure 2:**
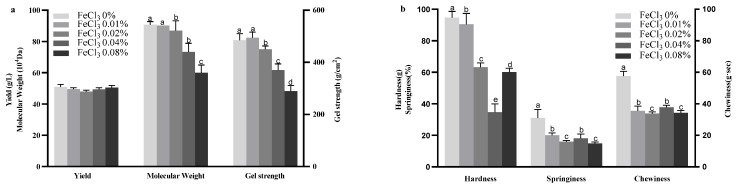
Effect of FeCl_3_ concentration on curdlan yield, gel strength, and molecular weight (Mw) (**a**), and Texture Profile Analysis (TPA) of curdlan gel (**b**). Different lowercase letters denote significant difference (*p* < 0.05).

The effects of varying concentrations of FeCl_3_ in the fermentation medium on curdlan yield, gel strength, and molecular weight are depicted in Figure 2. Figure 2 depicts the impact of FeCl_3_ concentration on curdlan production, gel strength, and molecular weight (Mw) (a), and Texture Profile Analysis (TPA) of curdlan gel (b). In the control group, after 144 h of fermentation, the curdlan yield was determined to be 51.00 ± 1.50 g/L. The addition of 0.02% FeCl_3_ reduced curdlan yield compared to the control, while other FeCl_3_ concentrations had no substantial impact on yield.

As the FeCl_3_ concentration increased, the gel strength of curdlan progressively decreased. The addition of 0.01% FeCl_3_ did not significantly affect gel strength compared to the control, but 0.08% FeCl_3_ resulted in the lowest gel strength, measuring 290.00 ± 21.50 g/cm^2^. This may be attributed to the different interfacial effects exerted by varying FeCl_3_ concentrations on gel strength, thus altering the stability of the gel. The low concentrations of iron ions can chelate with extracellular polysaccharides, thereby stabilizing the network structure and enhancing gel formation. However, owing to the strong oxidative nature of iron ions, higher FeCl_3_ concentrations may trigger oxidative reactions within the extracellular polysaccharides. This oxidation can lead to the degradation of the polysaccharides, thereby compromising the stability of the gel structure [34]. A similar trend was observed for molecular weight, with 0.02%, 0.04%, and 0.08% FeCl_3_ significantly reducing molecular weight to values of 86.95 ± 6.23 × 10^4^ Da, 73.28 ± 5.58 × 10^4^ Da and 60.12 ± 5.00 × 10^4^ Da, respectively. These findings highlight the detrimental effects of higher FeCl_3_ concentrations on curdlan’s structural and functional properties.

The TPA of curdlan gel produced with varying concentrations of FeCl_3_ in the fermentation medium for curdlan production is shown in Figure 2. The Figure depicts the influence of FeCl_3_ concentration on curdlan production, gel strength, and molecular weight (Mw) (a), and Texture Profile Analysis (TPA) of curdlan gel (b). The addition of 0.01% FeCl_3_ slightly influenced the hardness of the gel but significantly reduced its springiness and chewiness. As reported, the addition of low concentrations of inorganic salt ions can enhance the hardness and water-holding capacity of emulsion gels [35]. Higher concentrations of FeCl_3_ (0.02%, 0.04%, and 0.08%) had pronounced negative effects on all three parameters, hardness, springiness, and chewiness. As an oxidizing agent, FeCl_3_ can break the glycosidic bonds in the curdlan molecules, leading to chain cleavage and ultimately affecting its texture properties [36]. These findings suggest that FeCl_3_ is unsuitable as an additive in the fermentation medium for curdlan production, as it adversely impacts the gel’s textural properties.

### 2.2. Effect of FeSO_4_ on Curdlan Production and Properties

**Figure 3:**
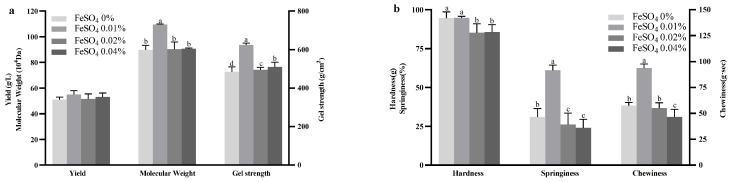
Effect of FeSO_4_ concentration on curdlan yield, gel strength, and molecular weight (Mw) (**a**), and Texture Profile Analysis (TPA) of curdlan gel (**b**). Different lowercase letters denote significant difference (*p* < 0.05).

The effects of varying FeSO_4_ concentrations in the fermentation medium on curdlan yield, gel strength and MW are presented in Figure 3. The addition of 0.01% FeSO_4_ resulted in the highest curdlan yield of 55.00 ± 3.00 g/L, compared to the control group’s yield of 51.00 ± 2.00 g/L. Interestingly, the addition of 0.01% FeSO_4_ led to significantly higher gel strength and molecular weight compared to other treatment groups, increasing gel strength from 485.00 ± 25.00 g/cm^2^ (control) to 624.16 ± 8.93 g/cm^2^ and molecular weight from 89.79 ± 3.59 × 10^4^ Da (control) to 109.502 ± 0.55 × 10^4^ Da. du Poset et al. reported that the Fe^2+^ ions serve as ionic cross-linking agents in galacturonate hydrogels, helping explain the observed increase in curdlan gel strength [37]. The addition of 0.02% FeSO_4_ had no effect on curdlan yield, whereas 0.04% FeSO_4_ increased curdlan yield slightly. Both 0.02% and 0.04% FeSO_4_ had no impact on molecular weight, but they slightly influenced gel strength. This phenomenon is consistent with the literature reports that FeSO_4_ can act as a catalyst, accelerating the decomposition of H_2_O_2_ to produce hydroxyl radicals (•OH), which in turn trigger the oxidative degradation of β-glucans, reducing their molecular weight and thereby affecting their gelation ability. It has also been noted that there are exceptions: the addition of low concentrations of FeSO_4_ results in shorter and more flexible polymer chains, which can facilitate the development of a more stable cross-linked structure and enhance gel strength. This finding is in agreement with the results presented in Figure 3 [38].

The TPA results for curdlan gels with varying concentrations of FeSO_4_ are depicted in Figure 3. The addition of 0.01% FeSO_4_ significantly enhanced the gel’s springiness (60.95 ± 3.41%) and chewiness (93.55 ± 3.94 g·s) (*p* < 0.05) without affecting hardness (*p* > 0.05). In contrast, 0.04% FeSO_4_ reduced hardness, springiness, and chewiness, while 0.02% FeSO_4_ lowered hardness and springiness but had no effect on chewiness. These results demonstrate that incorporating 0.01% FeSO_4_ in the fermentation medium can improve the springiness and chewiness of curdlan gels, offering an optimized approach for enhancing their textural properties.

### 2.3. Effect of MnCl_2_ on Curdlan Production and Properties

**Figure 4:**
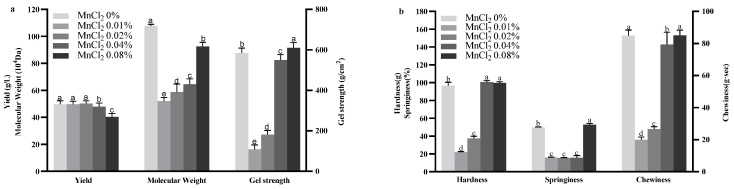
Effect of MnCl_2_ concentration on curdlan yield, gel strength, and molecular weight (Mw) (**a**), and Texture Profile Analysis (TPA) of curdlan gel (**b**). Different lowercase letters denote significant difference (*p* < 0.05).

The effects of varying concentrations of MnCl_2_ in the fermentation medium on curdlan yield, gel strength, and Mw are depicted in Figure 4. In contrast to the control group, the inclusion of 0.04% and 0.08% MnCl_2_ reduced the curdlan yield to 48.00 ± 2.65 g/L and 40.28 ± 2.54 g/L, respectively, while 0.01% and 0.02% MnCl_2_ had no significant impact on yield. Gel strength decreased notably with the addition of 0.01% and 0.02% MnCl_2_, dropping to 109.24 ± 21.30 g/cm^2^ and 181.60 ± 20.40 g/cm^2^, respectively, whereas 0.08% MnCl_2_ led to an increase in gel strength. This phenomenon may be attributed to the fact that Mn^2+^ possesses high coordination ability. Mn^2+^ interacts with metabolic product molecules through cross-linking to form stable complexes [39]. High concentrations of Mn^2+^ can increase the cross-linking density without causing excessive cross-linking or precipitation due to high charge, thereby maintaining favorable gel properties [40]. The molecular weight of curdlan significantly declined with 0.01%, 0.02%, and 0.04% MnCl_2_, reaching 51.99 ± 2.64 × 10^4^ Da, 58.87 ± 5.79 × 10^4^ Da, and 64.52 ± 3.96 × 10^4^ Da, respectively, compared to the molecular weight of control group at 107.90 ± 1.00 × 10^4^ Da. These findings suggest that MnCl_2_ concentration significantly influences curdlan’s yield, gel strength, and molecular weight, with higher concentrations adversely affecting molecular weight while showing a variable impact on gel strength.

The TPA results for curdlan gels with varying MnCl_2_ concentrations are summarized in Figure 4. Compared to the control, the inclusion of 0.01% and 0.02% MnCl_2_ considerably reduced the hardness, springiness, and chewiness of the gels. In contrast, the addition of 0.04% MnCl_2_ increased the hardness but decreased the chewiness. The addition of 0.08% MnCl_2_ further enhanced both hardness and springiness, while it had no notable effect on the chewiness. These findings highlight the concentration-related effects of MnCl_2_ on the textural properties of curdlan gels.

### 2.4. Effect of CoCl_2_ on Curdlan Production and Properties

**Figure 5:**
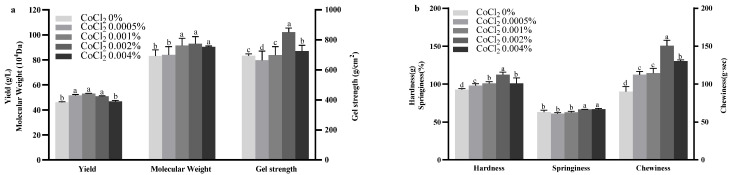
Effect of CoCl_2_ concentration on curdlan yield, gel strength, and molecular weight (Mw) (**a**), and Texture Profile Analysis (TPA) of curdlan gel (**b**). Different lowercase letters denote significant difference (*p* < 0.05).

The effects of varying CoCl_2_ concentrations in the fermentation medium on curdlan yield, gel strength, and molecular weight are illustrated in Figure 5. The addition of 0.0005%, 0.001%, and 0.002% CoCl_2_ significantly increased curdlan production, with the highest yield (53.15 ± 0.15 g/L) observed at 0.001% CoCl_2_. In contrast, 0.004% CoCl_2_ had no significant effect on yield. Regarding gel strength and Mw, the addition of 0.001%, 0.002%, and 0.004% CoCl_2_ significantly improved both parameters. The highest gel strength, 850.27 ± 29.05 g/cm^2^, was achieved with 0.002% CoCl_2_, which also corresponded to the highest molecular weight of 92.93 ± 5.67 × 10^4^ Da. These findings indicate that incorporating 0.002% CoCl_2_ in the fermentation medium optimizes curdlan gel strength and molecular weight, enhancing its overall quality.

The TPA results for curdlan gels with varying concentrations of CoCl_2_ are depicted in Figure 5. In juxtaposition with the control, both hardness and chewiness rose with the addition of CoCl_2_, peaking at 0.002% CoCl_2_ (hardness: 112.38 ± 3.43 g; chewiness: 150.69 ± 7.08 g·s), before declining at higher concentrations, such as 0.004%. Additionally, the springiness of curdlan gels improved with the addition of 0.002% and 0.004% CoCl_2_. Previous studies have also reported that increasing Co^2+^ concentrations can induce a conformational shift in extracellular polysaccharides (EPS), transitioning from a loose spherical structure to a more compact and highly ordered helical form [41]. These results indicate that incorporating CoCl_2_ at appropriate concentrations enhances the textural properties of curdlan gels, with 0.002% CoCl_2_ providing the optimal effect.

### 2.5. Effect of ZnCl_2_ on Curdlan Production and Properties

**Figure 6:**
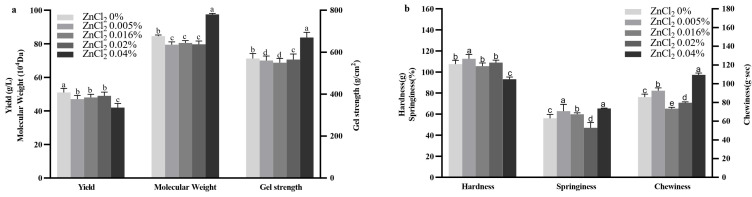
Effect of ZnCl_2_ concentration on curdlan yield, gel strength, and molecular weight (Mw) (**a**), and Texture Profile Analysis (TPA) of curdlan gel (**b**). Different lowercase letters denote significant difference (*p* < 0.05).

The effects of varying ZnCl_2_ concentrations in the fermentation medium on curdlan yield, gel strength, and molecular weight are presented in Figure 6. While most ZnCl_2_ concentrations led to a reduction in curdlan yield, 0.02% ZnCl_2_ maintained the same yield as that of the control group. The lowest yield, 42.27 ± 2.41 g/L, occurred at a 0.04% ZnCl_2_. Despite this, 0.04% ZnCl_2_ resulted in the highest gel strength, 669.73 ± 25.40 g/cm^2^, and the highest molecular weight, 97.50 ± 0.61 × 10^4^ Da. Lower ZnCl_2_ concentrations slightly reduced both gel strength and molecular weight compared to the control group. These results suggest that 0.04% ZnCl_2_ effectively enhances the gel strength and Mw of curdlan, despite causing a modest reduction in yield.

The TPA results of curdlan gels with varying concentrations of ZnCl_2_ are depicted in Figure 6. In juxtaposition with the control, the addition of 0.005% ZnCl_2_ increased the hardness, springiness, and chewiness of curdlan gels. At a concentration of 0.04% ZnCl_2_ the springiness (65.38 ± 0.37%) and chewiness (109.43 ± 2.00 g·s) were enhanced, while the hardness decreased.

### 2.6. The Relationship Between Gel Strength and Molecular Weight

**Figure 7:**
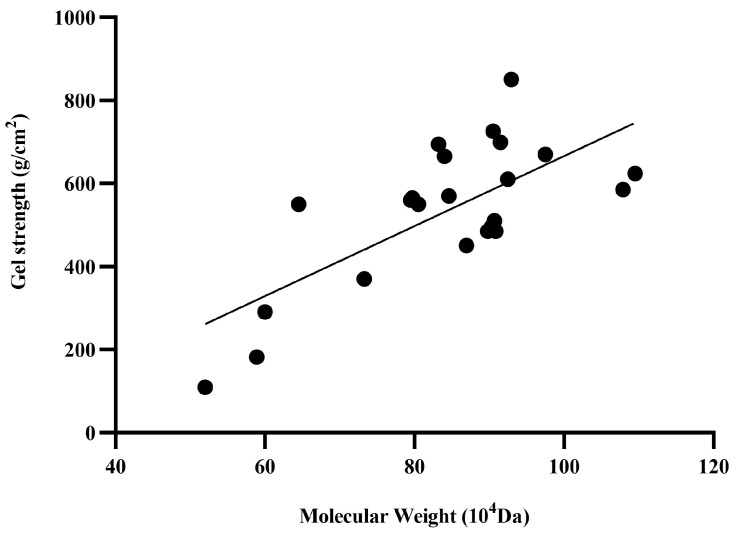
Correlation analysis results between molecular weight and gel strength.

The reciprocal relationship between gel strength and Mw was analyzed using all the above data (Figure 2, Figure 3, Figure 4, Figure 5 and Figure 6), as summarized in Figure 7. The molecular weight values were determined based on GPC curves of curdlan obtained under varying inorganic salt concentrations, shown in Figures S1–S5. A regression equation (Y = 8.430X − 177.1) was derived, with a correlation coefficient (R^2^) of 0.5064. This indicates a moderate linear relationship between molecular weight and gel strength. The regression demonstrated robust statistical significance (*p* < 0.0001), and the 95% confidence interval for the slope (4.75–12.11) excluded zero, further supporting the presence of a linear association. The changes in gel strength are not solely dependent on the magnitude of molecular weight but are also closely related to the uniformity of molecular weight distribution, cross-linking efficiency, and the interactions between polymer chains [42]. These findings suggest a preliminary relationship between gel strength and molecular weight; however, further experimentation is necessary to confirm the validity and robustness of this correlation.

## 3. Conclusions

This study systematically investigated the effects of various inorganic salts on curdlan production, gel strength, molecular weight (Mw), and texture properties, identifying key trends and optimal conditions. FeCl_3_ negatively impacted all measured parameters, with higher concentrations degrading glycosidic bonds and reducing gel quality, making it unsuitable for curdlan fermentation. FeSO_4_ at 0.01% significantly improved yield, gel strength, and texture properties such as springiness and chewiness, but higher concentrations were detrimental. MnCl_2_ showed minimal effects on yield, but low concentrations reduced gel strength, hardness, springiness, and chewiness. CoCl_2_ emerged as the most effective additive, with 0.002% significantly enhancing curdlan yield, Mw, gel strength, and texture properties, though higher levels reduced these benefits. ZnCl_2_ had minimal effects on yield and general texture but improved chewiness, gel strength, and Mw at 0.04%. A proportional relationship between Mw and gel strength (R^2^ = 0.5064) suggests Mw is critical to curdlan’s gel strength. Overall, the study provides valuable insights into optimizing inorganic salt concentrations to enhance curdlan quality, highlighting CoCl_2_ and FeSO_4_ as promising additives for industrial applications.

## 4. Materials and Methods

### 4.1. Materials

All chemical reagents used in the context of this research were purchased from Sinopharm Chemical Reagents Co., Ltd. (Shanghai, China) [43], comprising organic substrates (sucrose, glycerol, urea), nitrogen sources (peptone, tryptone, soybean peptone, beef extract, yeast extract), and inorganic salts [(NH_4_)_2_HPO_4_, NH_4_Cl, (NH_4_)_2_SO_4_, NaNO_3_, KH_2_PO_4_, MgSO_4_·7H_2_O, CaCO_3_]. Corn syrup was sourced from Angel Yeast Co., Ltd., located in Hubei, China. High-purity pullulan and dextran standards were obtained from Malvern Instruments Ltd., Malvern, UK.

### 4.2. Microorganisms

The *Agrobacterium* sp. CGMCC 11546 was preserved at −80 °C in 20% glycerol-enriched (*v*/*v*) Luria−Bertani (LB) medium (East China Normal University, Shanghai, China).

### 4.3. Mediums

The seed medium for culturing *Agrobacterium* sp. CGMCC 11546 comprised the following (%, *w*/*v*): 2.0 sucrose, 0.5 (NH_4_)_2_HPO_4_, 0.15 KH_2_PO_4_, 0.1 MgSO_4_·7H_2_O, 0.3 corn syrup, and 0.3 CaCO_3_ [44]. The fermentation medium consisted of (%, *w*/*v*): 9.0 sucrose, 0.2 (NH_4_)_2_HPO_4_, 0.2 KH_2_PO_4_, 0.1 MgSO_4_·7H_2_O. Both media were regulated to pH 7.0 prior to autoclaving (121 °C, 15 min).

### 4.4. Culture Conditions

For preparing seed cultures of *Agrobacterium* sp. CGMCC 11546, glycerol-based culture stocks stored at −80 °C were aseptically transferred into 50 mL of seed medium (250 mL Erlenmeyer flask) and incubated at 30 °C with orbital agitation (250 rpm) for 20 h. A 5% volume proportion aliquot of the primary medium was transferred to fresh seed culture (50 mL/250 mL flask) under identical conditions for 16 h to amplify biomass. Fermentation was initiated by inoculating 10% (*v*/*v*) of the secondary culture into 50 mL production medium (500 mL flask), followed by 96 h of incubation (30 °C, 250 rpm) [45].

### 4.5. Effect of Inorganic Salt Ions on the Gel Strength of Curdlan

In the fermentation experiment, five distinct inorganic salts, namely FeCl_3_, FeSO_4_, MnCl_2_, CoCl_2_, and ZnCl_2_, were selected for evaluation. Their concentrations were selected based on two references [17,46]. These inorganic salts were applied at the following concentrations (*w*/*v*): FeCl_3_ at 0.01%, 0.02%, 0.04%, and 0.08%; FeSO_4_ at 0.01%, 0.02%, and 0.04%; MnCl_2_ at 0.01%, 0.02%, 0.04%, and 0.08%; CoCl_2_ at 0.0005%, 0.001%, 0.002%, and 0.004%; and ZnCl_2_ at 0.005%, 0.016%, 0.02%, and 0.04%. A control group, devoid of inorganic salts, was included. After a 96-h fermentation period, the curdlan yield, gel strength, and molecular weight were measured to evaluate the impact of inorganic salt ions on gel strength.

### 4.6. Measurement of Curdlan Production

Curdlan production was quantified using a modified acid–alkali precipitation protocol [47]. Briefly, fermentation broth (5 mL) was mixed with 0.5 N NaOH (100mL) at 60 °C for 1 h to solubilize curdlan. The mixture was centrifuged (8000× *g*, 10 min), and the supernatant acidified with 0.5 N HCl (100 mL) to precipitate curdlan. The precipitate was collected by centrifugation (8000× *g*, 10 min), washed thrice with deionized water to eliminate ionic residues, and dried to constant weight at 60 °C.

### 4.7. Curdlan Purification

Curdlan purification was implemented as outlined by Gao et al. [17]. Initially, the fermentation broth was mixed with 1 M NaOH (1:1, *v*/*v*) under vigorous agitation (10 min), followed by static incubation (1 h) to ensure complete dissolution. Cellular debris was removed by centrifugation (8000× *g*, 30 min, 4 °C). The resulting supernatant underwent acid-induced precipitation (3 M HCl) and filtration (120-mesh filter cloth). The precipitate was further purified by washing thrice with deionized water, followed by rinsing with dehydrated ethanol at three times the volume of the precipitate. Finally, the product was freeze-dried. The purified samples were subsequently analyzed for gel strength and molecular weight.

### 4.8. Gel Strength and TPA Determination

Curdlan gel strength, defined as the maximum force a gel can withstand before deformation or structural failure under applied force, was determined using a TA-XT plus texture analyzer (Stable Micro System, Surrey, UK) fitted with a P/0.5 cylindrical probe. Gel samples were prepared according to a previously established method [48]. A 15 mL aliquot of 2% purified curdlan solution was evenly dispensed into glass tube (18 mm diameter × 180 mm height), degassed for 4 min using a circulating water vacuum pump, and thermally treated in a water bath at 95 °C for 10 min, followed by rapid cooling in a 4 °C cold-water bath. The resulting gel was horizontally sliced 20–35 mm from the bottom. Gel strength was measured via the penetration method using a P/5 cylindrical stainless-steel probe (5 mm diameter). The testing parameters were set as follows: pretest speed of 2.0 mm/s, test speed of 1.0 mm/s, post-test speed of 2.0 mm/s, test distance of 10 mm, and a trigger force of 10.0 g.

The textural properties of curdlan gel were also analyzed using the texture analyzer with slight modifications to a previously established method [49]. Gel samples of uniform dimensions (10 mm height × 16mm diameter) were compressed twice to 50% of their original height using a P/50 cylindrical probe, with a 10 s rest between cycles. Testing parameters were set with pre-test, test, and post-test speeds of 2.0, 1.0, and 2.0 mm/s, respectively, a deformation (strain) level of 50%, and a trigger force of 10 g. The resulting force-time curves were analyzed using the Texture Exponent software (Version 6,1,26,0) to evaluate hardness, springiness, gumminess, cohesiveness, and chewiness.

### 4.9. Molecular Weight (MW) Determination

Curdlan’s average molecular weight (Mw) was analyzed using gel permeation chromatography coupled with multi-angle light scattering (GPC-MALLS), performed on a Malvern Viscotek TDAmax system equipped with refractive index (RI) and light scattering (LS) detectors. The method was adapted from Szwengiel and Wiesner [50] using high-performance gel permeation chromatography. According to Rayleigh scattering principles, as applied by Wyatt, LS intensity in dilute polymer solutions exhibits a linear relationship with the product of Mw [51]. In this study, a pullulan standard (Mw: 72,812 Da; polydispersity index (PDI) = 1.07) was employed for column calibration, and a dextran standard (73,253 Da; PDI = 1.25) was used to validate chromatographic resolution. Lyophilized curdlan was dissolved in dimethyl sulfoxide (DMSO) under continuous thermal agitation (100 °C, 4 h). The homogenized solution (0.5 mg/mL) was chromatographed using an I-MBHMW-3078 polar organic column (7.8 × 300 mm, 50 °C) with isocratic elution (DMSO mobile phase, 0.5 mL/min), employing a 40 μL injection volume. The average Mw was calculated using Omni SEC 5.12 software (Malvern, UK) by integrating LS and RI detector data, following the approach described by Deng et al. [52]. The molecular weight reported in this study refers specifically to the average molecular weight (Mw).

### 4.10. Statistical Analysis

Statistical significance was evaluated using one-way ANOVA with SPSS version 26 (IBM, Armonk, NY, USA). Results were presented as means ± standard deviation (SD) from a minimum of three experiments. For one-way ANOVA, the Tukey ’s HSD tests were performed to compare means across multiple groups.

## Data Availability

The data that support the findings of this study area available from the corresponding authors upon reasonable request.

## Supplementary Materials

The following supporting information can be downloaded at: https://www.mdpi.com/article/10.3390/gels11050313/s1. Figure S1: GPC curves of curdlan obtained under varying FeCl_3_ concentrations; Figure S2: GPC curves of curdlan obtained under varying FeSO_4_ concentrations; Figure S3: GPC curves of curdlan obtained under varying MnCl_2_ concentrations; Figure S4: GPC curves of curdlan obtained under varying CoCl_2_ concentrations; Figure S5: GPC curves of curdlan obtained under varying ZnCl_2_ concentrations.
