# Supplementary material for: Effects of Inorganic Salts on Curdlan Production and Structural Properties
Source: Gels. 2025 Apr 23;11(5):313. doi: 10.3390/gels11050313 (PMC12111470; doi:10.3390/gels11050313)
Supplement: Supplementary file 1 [file gels-11-00313-s001.zip › gels-3574857-supplementary.pdf]

Effects of Inorganic Salts on Curdlan Production and Structural Properties

Xinyi Zhu [1,2,†], Bowei Yao [3,*,†], Siyang Yue [2], Zhongyi Chang [2], Xuexia Yang [1,*] and Hongliang Gao [2]

[1] College of Biological Science and Medical Engineering, Donghua University, Shanghai 201620, China;
zhuxinyi0820@163.com
[2] School of Life Sciences, East China Normal University, Shanghai 200241, China;
siyangyue2000@163.com (S.Y.); zychang@bio.ecnu.edu.cn (Z.C.); hlgao@bio.ecnu.edu.cn (H.G.)
[3] NingXia Academy of Metrology & Quality Inspection, Yinchuan 750001, China
* Correspondence: yaobowei@hotmail.com (B.Y.); yxx@dhu.edu.cn (X.Y.)
† These authors contributed equally to this work.

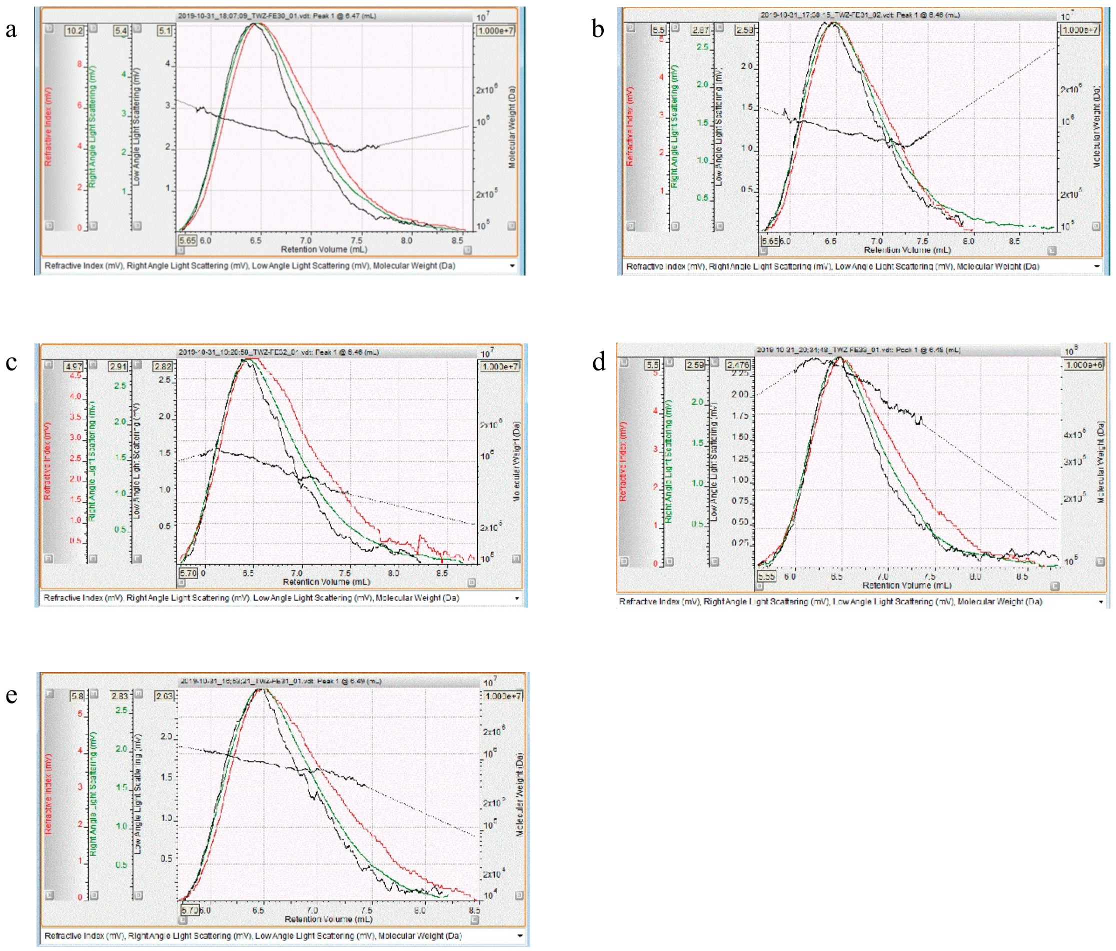

Figure S-1. GPC curves of curdlan obtained under varying $FeCl_3$ concentrations. (a) 0% $FeCl_3$, (b) 0.01% $FeCl_3$, (c) 0.02% $FeCl_3$, (d) 0.04% $FeCl_3$, (f) 0.08% $FeCl_3$

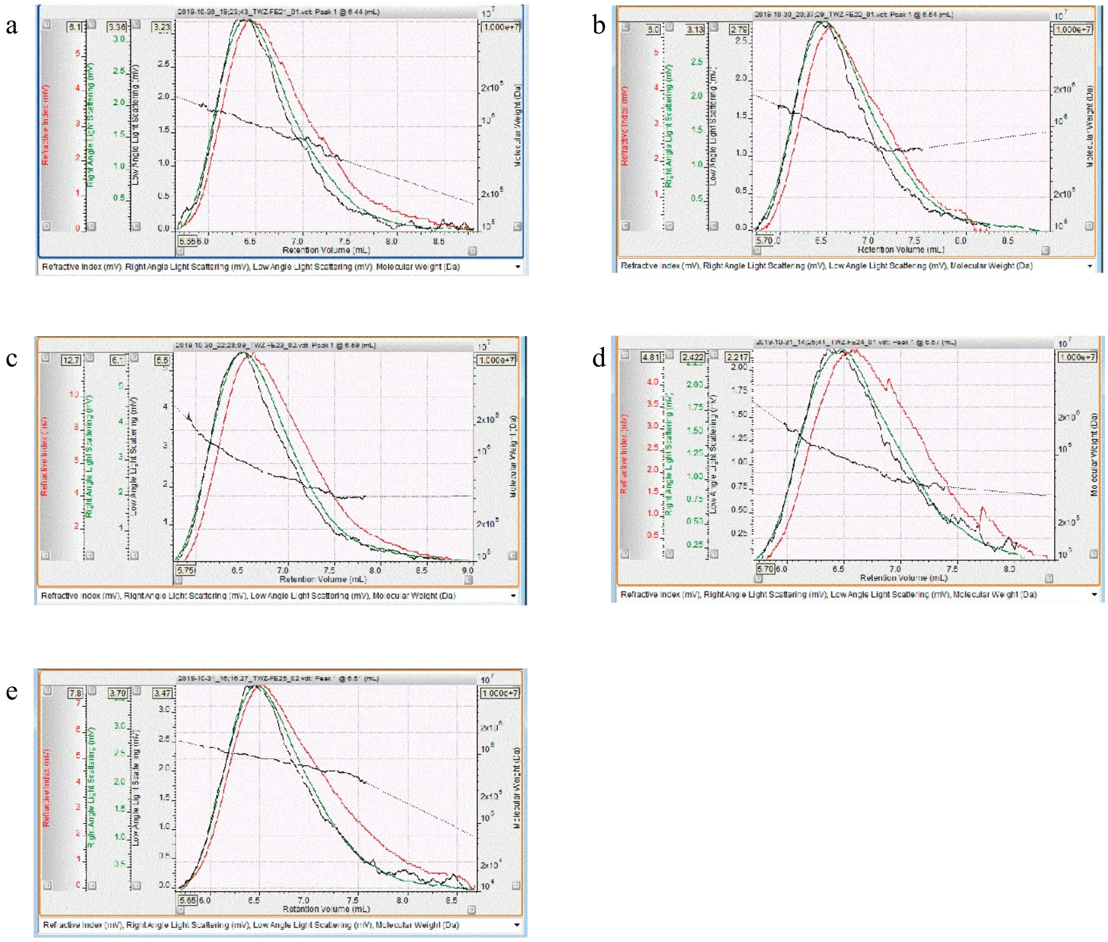

Figure S-2. GPC curves of curdlan obtained under varying $FeSO_4$ concentrations. (a) 0% $FeSO_4$, (b) 0.01% $FeSO_4$, (c) 0.02% $FeSO_4$, (d) 0.04% $FeSO_4$, (f)0.08% $FeSO_4$

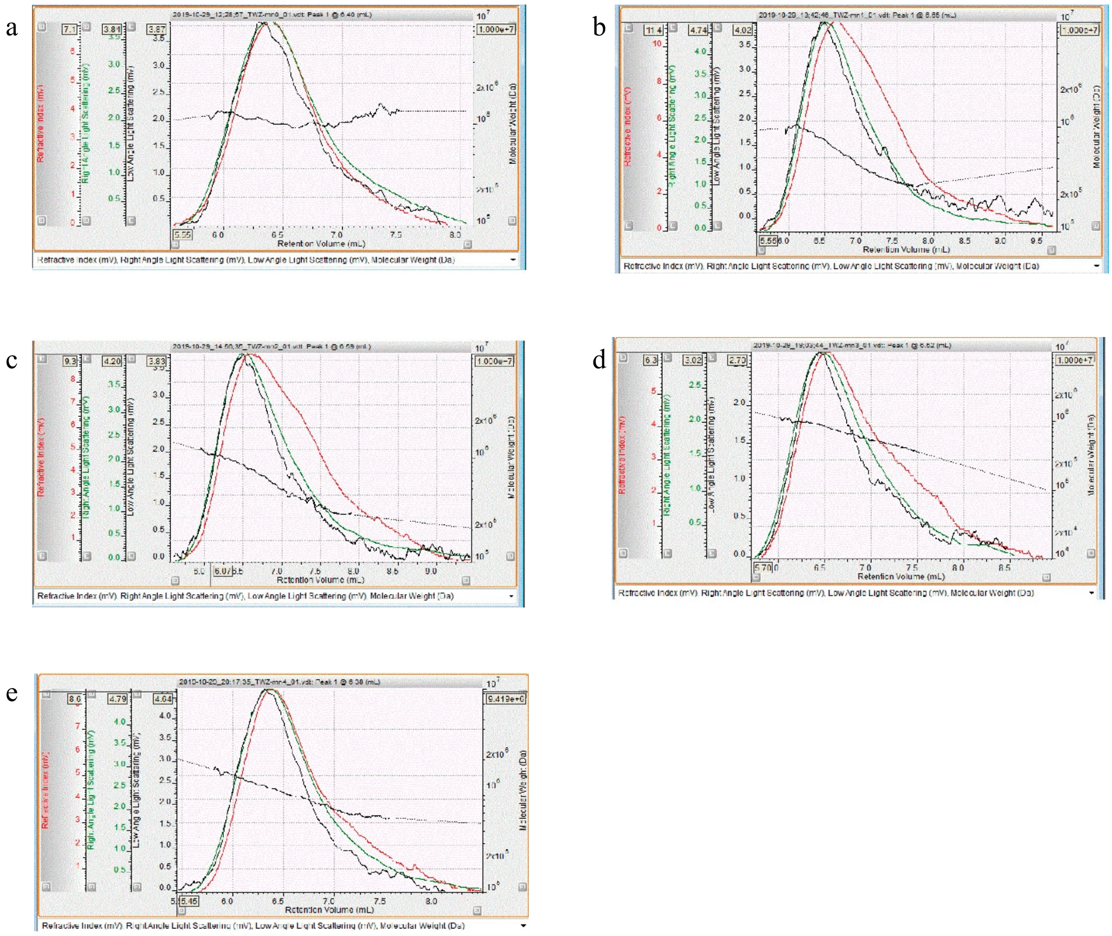

Figure S-3. GPC curves of curdlan obtained under varying $MnCl_2$ concentrations. (a) 0% $MnCl_2$, (b) 0.01% $MnCl_2$, (c) 0.02% $MnCl_2$, (d) 0.04% $MnCl_2$, (f)0.08% $MnCl_2$

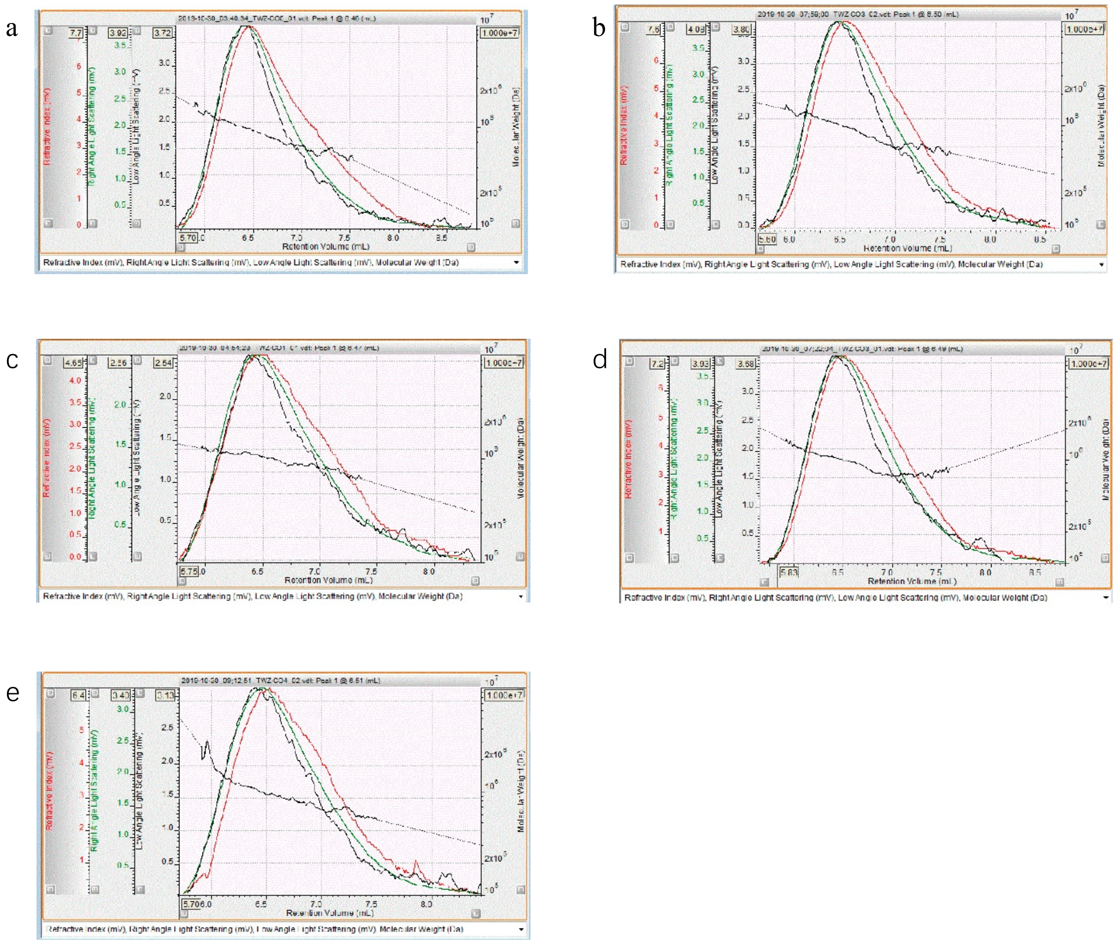

Figure S-4. GPC curves of curdlan obtained under varying $CoCl_2$ concentrations. (a) 0% $CoCl_2$, (b) 0.0005% $CoCl_2$, (c) 0.001% $CoCl_2$, (d) 0.002% $CoCl_2$, (f)0.004% $CoCl_2$

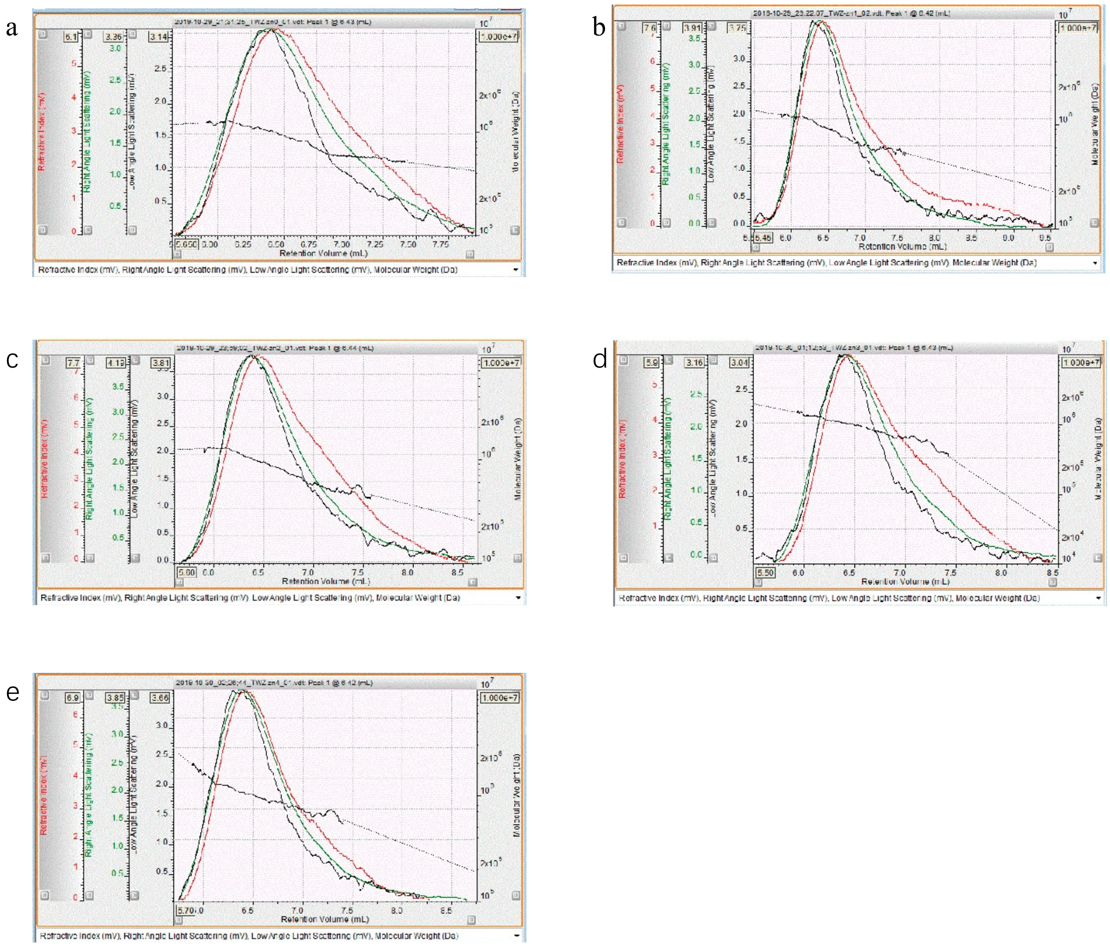

Figure S-5. GPC curves of curdlan obtained under varying $ZnCl_2$ concentrations. (a) 0% $ZnCl_2$, (b) 0.005% $ZnCl_2$, (c) 0.016% $ZnCl_2$, (d) 0.02% $ZnCl_2$, (f)0.04% $ZnCl_2$
